# Intratumoral injection of STING ligand promotes abscopal effect

**DOI:** 10.1186/2051-1426-2-S3-P158

**Published:** 2014-11-06

**Authors:** Jason R Baird, Thomas W Dubensky, David B Kanne, David J Friedman, Benjamin Cottam, Shelly Bambina, Keith S Bahjat, Marka Crittenden, Michael Gough

**Affiliations:** 1 Earle A. Chiles Research Institute, Robert W. Franz Cancer Center, Providence Portland Medical Center, Portland, OR, USA; 2 Aduro Biotech, Berkeley, CA, USA; 3 The Oregon Clinic, Portland, OR, USA

## 

The abscopal effect is a phenomenon that occurs when radiation of disease at one-site leads to regression of distant, untreated tumors. Historically, the abscopal effect caused by conventional radiation therapy has been rare. However, the combination of radiation and immunotherapy has increased the reported incidence of this effect, which is thought to be mediated by an adaptive immune response. We have demonstrated that radiation alone (RT) is weakly effective at generating anti-tumor adaptive immune responses despite release of tumor-associated antigen. We hypothesized that providing a potent adjuvant to the tumor would improve the adaptive immune response increasing immune control of local and distant disease The STimulator of INterferon Genes (STING) innate signaling pathway is being investigated for its ability to propagate an adaptive immune response that is initiated in the tumor microenvironment (TME). STING is a cytosolic receptor that senses either exogenous or endogenous cyclic dinucleotides (CDNs), produced by bacteria or by host cell cyclic GMP-AMP synthetase in response to binding dsDNA, respectively. In the current work, we evaluated synthetic CDN derivatives modified to be resistant to degradation by phosphodiesterases and to activate all known human STING allele variants. To test our hypothesis, we established bilateral Panc02 flank tumors in immune competent C57BL/6 mice. 14 days post tumor challenge mice were randomized to receive a suboptimal dose of 10 Gray of RT to one tumor, and intratumoral injection of CDN or vehicle to that same tumor immediately following radiation and again at 24 hours. CDN injection resulted in rapid local and systemic induction of inflammatory mediators including IFNγ and TNFα (n = 5, p < 0.01), and vascular damage that spread through the injected tumor without causing detectable damage to normal tissues. CDN injection caused rapid innate-mediated regression of injected tumors (n = 15, p < 0.001), and when combined with local radiation therapy also caused adaptive-immune mediated control of distant tumors (n = 12, p < 0.01). We conclude that these CDNs are potent activators of innate immunity that in this model combine effectively with radiation therapy to promote adaptive immune activation and systemic immune surveillance.

